# Induction of glioblastoma cell ferroptosis using combined treatment with chloramphenicol and 2-deoxy-d-glucose

**DOI:** 10.1038/s41598-023-37483-5

**Published:** 2023-06-28

**Authors:** Kenji Miki, Mikako Yagi, Naoki Noguchi, Yura Do, Ryosuke Otsuji, Daisuke Kuga, Dongchon Kang, Koji Yoshimoto, Takeshi Uchiumi

**Affiliations:** 1 Department of Clinical Chemistry and Laboratory Medicine, Graduate School of Medical Sciences, Kyushu University, Higashi-Ku, Fukuoka, 812-8582 Japan; 2 Department of Neurosurgery, Graduate School of Medical Sciences, Kyushu University, Higashi-Ku, Fukuoka, 812-8582 Japan; 3 Department of Health Sciences, Graduate School of Medical Sciences, Kyushu University, Higashi-Ku, Fukuoka, 812-8582 Japan

**Keywords:** CNS cancer, Cell death, Transcription, Translation

## Abstract

Glioblastoma, a malignant tumor, has no curative treatment. Recently, mitochondria have been considered a potential target for treating glioblastoma. Previously, we reported that agents initiating mitochondrial dysfunction were effective under glucose-starved conditions. Therefore, this study aimed to develop a mitochondria-targeted treatment to achieve normal glucose conditions. This study used U87MG (U87), U373, and patient-derived stem-like cells as well as chloramphenicol (CAP) and 2-deoxy-d-glucose (2-DG). We investigated whether CAP and 2-DG inhibited the growth of cells under normal and high glucose concentrations. In U87 cells, 2-DG and long-term CAP administration were more effective under normal glucose than high-glucose conditions. In addition, combined CAP and 2-DG treatment was significantly effective under normal glucose concentration in both normal oxygen and hypoxic conditions; this was validated in U373 and patient-derived stem-like cells. 2-DG and CAP acted by influencing iron dynamics; however, deferoxamine inhibited the efficacy of these agents. Thus, ferroptosis could be the underlying mechanism through which 2-DG and CAP act. In conclusion, combined treatment of CAP and 2-DG drastically inhibits cell growth of glioblastoma cell lines even under normal glucose conditions; therefore, this treatment could be effective for glioblastoma patients.

## Introduction

Glioblastoma is one of the most malignant brain tumors with a poor prognosis and no curative treatment^1–3^. Temozolomide is a standard treatment for glioblastoma; however, it prolongs survival by only 2.5 months^3^. Furthermore, glioblastoma cells become resistant to temozolomide, and the mitochondria are associated with this resistance^3–5^.

The mitochondria are important in adenosine triphosphate production, calcium dynamics, β-oxidation, and reactive oxygen species generation^6^. Mitochondria are also associated with various diseases, including malignant tumors, especially in cancer stem cells^7,8^. Thus, we investigated a mitochondria-targeted treatment for glioblastoma.

Our previous study found that antimicrobial agents, including chloramphenicol (CAP) and doxycycline, are effective for treating glioblastoma under glucose-starved conditions because glioblastoma cells become dependent on the mitochondria in this condition^9^. A 3-day administration of these antimicrobial agents was ineffective in normal or high glucose concentrations^9^. Consequently, in this study, we aimed to develop an effective treatment for glioblastoma in conditions of normal glucose concentration.

2-Deoxy-d-glucose (2-DG), a glucose analog, inhibits hexokinase and phosphoglucose isomerase activities, blocking glycolysis^10^. Moreover, 2-DG mimics glucose-starved conditions, induces mitochondrial oxidative stress, and inhibits cancer cell growth^10^. Although Singh et al.^11^ reported combined treatment with 2-DG and radiotherapy for glioblastoma treatment, this approach is not used in clinical practice. However, 2-DG was used in some clinical trials for cancers or solid tumors^12,13^.

In the tumor environment, cells exist in glucose-starved and hypoxic conditions, which should be considered in the development of treatments for glioblastoma^14,15^. In this study, treatment efficacy was investigated in both normal and hypoxic conditions. In addition, results from cell line analysis were validated using stem-like cells mimicking an in vivo condition.

The mechanisms underlying cell death caused by each agent vary, including apoptosis, necroptosis, and ferroptosis^9,16^. Ferroptosis is a form of cell death that is currently attracting attention. In our previous study, CAP resulted in cell death via ferroptosis under glucose-starved conditions^9^. Ferroptosis has various markers, including FTH1, GpX4, KEAP1, and NRF2. Additionally, mRNA, PTGS2, CHAC1, and HO-1 are related to ferroptosis^17^. One of the inhibitors of the ferroptosis pathway is deferoxamine (DFO)^9^.

This study aimed to investigate the efficacy of a mitochondria-targeted treatment under a normal glucose condition. We examined the efficacy of CAP and 2-DG individually and combined. In addition, we investigated the mechanisms underlying cell death caused by these agents.

## Results

### Chloramphenicol (CAP) with glucose control is effective in the long term

**Figure 1:**
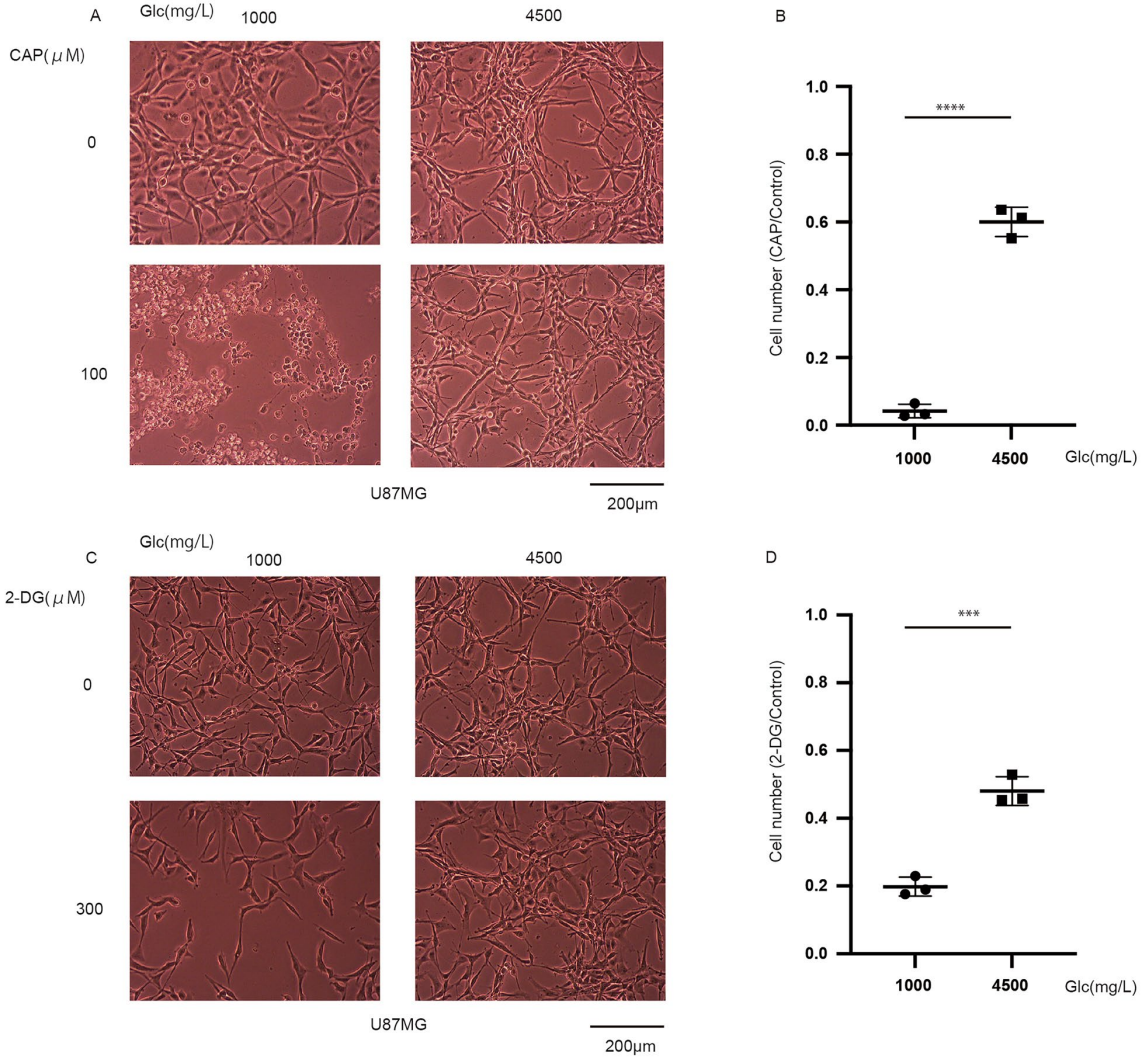
Effects of chloramphenicol (CAP) and 2-deoxy-d-glucose (2-DG) under different glucose conditions. (**a**) The effect of the 7-day administration of CAP in U87 (2 × 10^4^ cells were seeded) under normal (1000 mg/L) and high (4500 mg/L) glucose conditions. CAP is effective under normal glucose conditions. (**b**) Cell number with CAP under glucose concentrations of 1000 and 4500 mg/L relative to the control shows that CAP is effective under normal glucose conditions (cells were seeded in a six-well plate and counted using a Coulter counter). (**c**) The effect of the 3-day administration of 2-DG in U87 under normal and high glucose conditions; 2-DG is effective at 1,000 mg/L glucose condition. (**d**) Cell number with 2-DG under normal and high glucose concentrations relative to the control shows that 2-DG is effective under normal glucose conditions (cells were seeded in a six-well plate and counted using trypan blue). Values are presented as mean ± standard deviation. Student’s *t*-test was performed under normal glucose vs. high glucose conditions. ***P < 0.001, ****P < 0.0001.

In our previous study, CAP was only effective under glucose-starved conditions within 3 days and not under normal or high glucose conditions^9^. However, oxygen consumption rate (OCR) data revealed that oxidative phosphorylation (OXPHOS) was more activated in normal glucose concentrations than in high glucose concentrations^9^. Considering this result, we investigated the effect of long-term CAP administration. We observed that the 7-day administration of CAP was more effective under normal glucose conditions than under high glucose conditions (Fig. 1a,b). Although cells died in normal glucose concentrations, this effect was decreased in high glucose concentrations, highlighting the importance of glucose control during treatment.

We subsequently used 2-DG because it inhibits glycolysis, mimicking glucose-starved conditions^10^. Moreover, 2-DG has been considered for treating cancers and solid tumors^12,13^. We expected 2-DG to inhibit cell growth alone or in combination with CAP. The 3-day administration of 2-DG decreased cell numbers under normal glucose concentrations (Fig. 1c,d). The effect of this drug was also decreased under high glucose conditions.

### Combined treatment of 2-DG and CAP is effective

**Figure 2:**
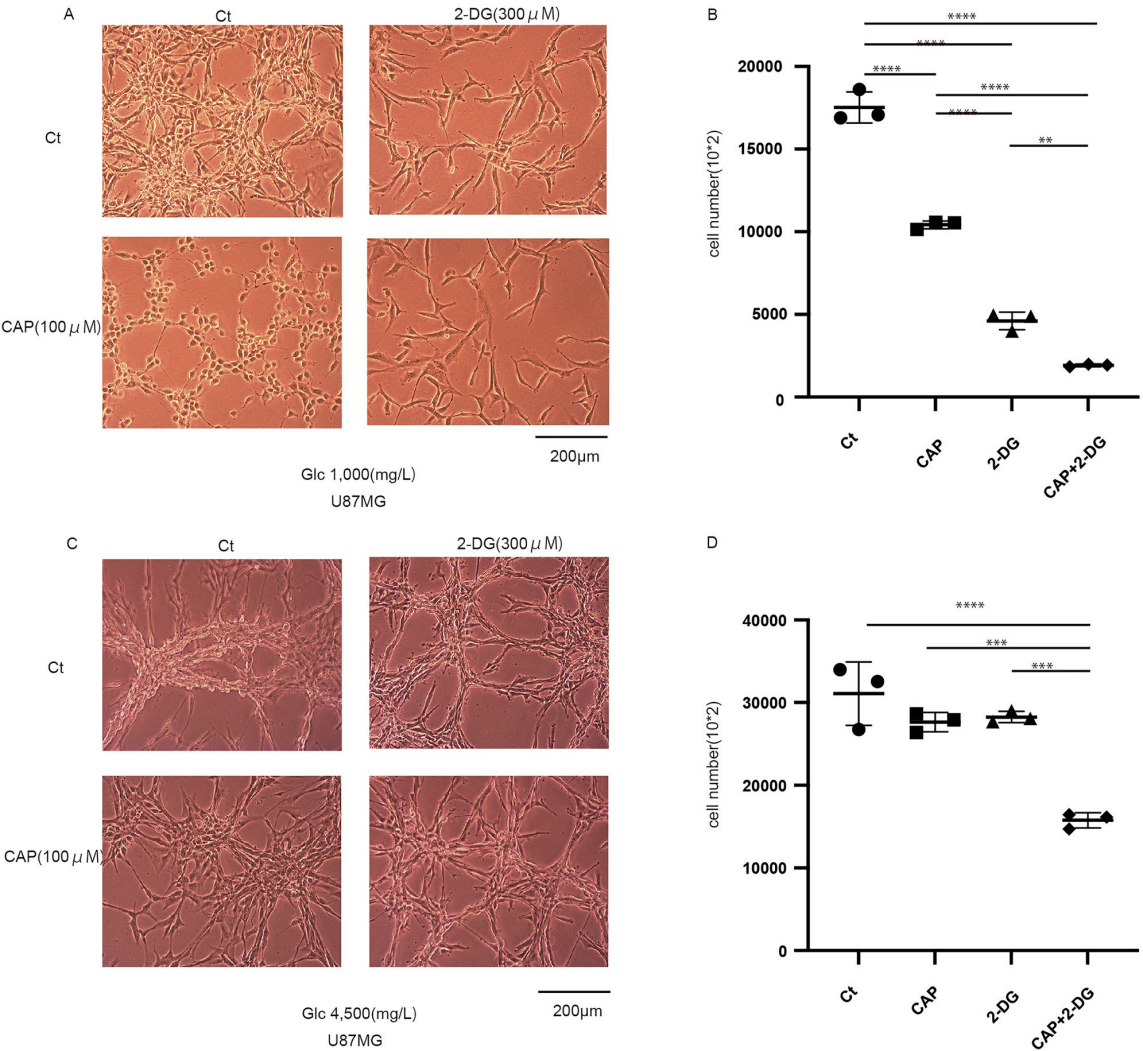
Effects of combined treatment, including chloramphenicol (CAP) and 2-deoxy-d-glucose (2-DG), under different glucose conditions. (**a**) The effect of the 5-day administration of CAP and 2-DG in U87 under normal glucose conditions. The combined treatment was effective. (**b**) Cell number with each agent under a glucose concentration of 1000 mg/L (cells were seeded in a six-well plate and counted using a Coulter counter). (**c**) The effect of the 5-day administration of CAP and 2-DG in U87 under high glucose conditions. (**d**) Cell number with each agent under a glucose concentration of 4500 mg/L (cells were seeded in a six-well plate and counted using a Coulter counter). Values are presented as mean ± standard deviation. Statistical significance was assessed using the ordinary one-way analysis of variance test with Tukey’s multiple comparison test assessing Ct vs. CAP vs. 2-DG vs. CAP + 2-DG. **P < 0.01, ***P < 0.001, ****P < 0.0001.

We hypothesized that the combined treatment of CAP and 2-DG is effective and, thus, assessed whether glioblastoma cells decreased after 3- and 5-day administration of these agents. Combined treatment was effective, and cell growth was inhibited in normal glucose conditions (Fig. 2a,b, Supplementary Fig. S1a–d). Moreover, cell count showed combination effects in CAP and 2-DG. However, under high glucose concentrations, the effectiveness of combined therapy decreased (Fig. 2c,d). This combined treatment is effective because 2-DG creates glucose-starved conditions.

### 2-DG increases COX1 expression and OXPHOS

**Figure 3:**
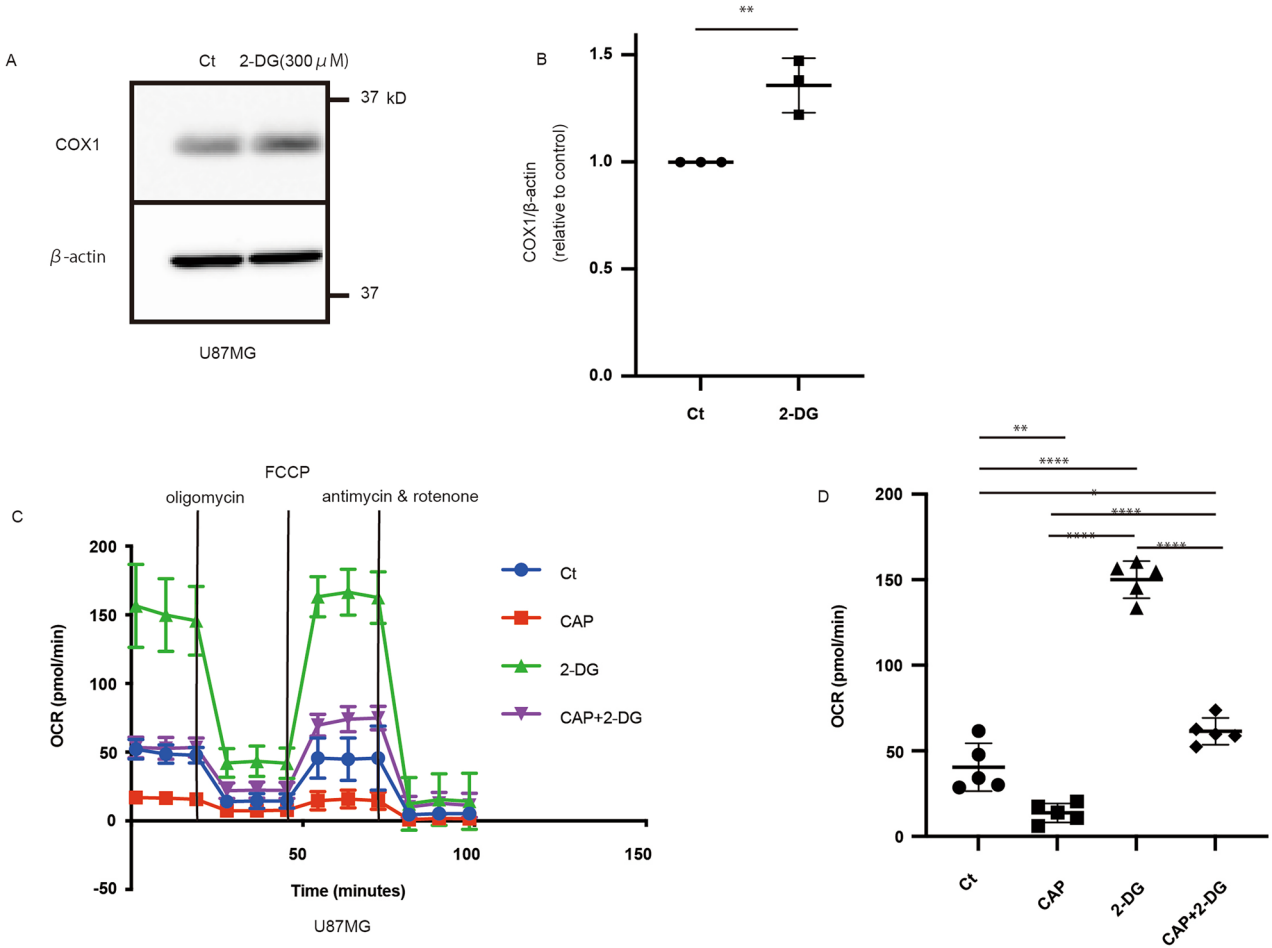
2-Deoxy-d-glucose (2-DG) makes the cell’s mitochondria dominant and increases the oxygen consumption rate (OCR). (**a**) 2-DG increased the expression of COX1 in U87 after 3 days. (**b**) Quantification of COX1 expression (N = 3). Values are presented as mean ± standard deviation. Student’s *t*-test was performed on normal glucose vs. high glucose conditions. **P < 0.01. (**c**) Traces of OCR in each agent administered and (**d**) quantification of maximal respiration. Ordinary one-way analysis of variance with Tukey’s multiple comparison test was performed to assess Ct vs. CAP vs. 2-DG vs. CAP + 2-DG. *P < 0.05, **P < 0.01, ****P < 0.0001.

COX1 expression increased after 2-DG administration (Fig. 3a,b). Therefore, we monitored the OCR to evaluate OXPHOS and found that the OCR was higher under 2-DG administration than the control and that the OCR decreased in the CAP group (Fig. 3c,d). Considering these results, 2-DG mimics glucose-starved conditions, increasing the effectiveness of CAP. Subsequently, we consider this nutrient-deprived condition leads to an increase in the activity of fatty acid oxidation to produce energy. To detect the OCR derived from fatty acid activation, etomoxir, a carnitine palmitoyltransferase 1A (CPT1A) inhibitor, was used^18,19^. OCR decreased when etomoxir was injected (Supplementary Fig. S2a,b).

### Combined treatment is effective even under hypoxic conditions

**Figure 4:**
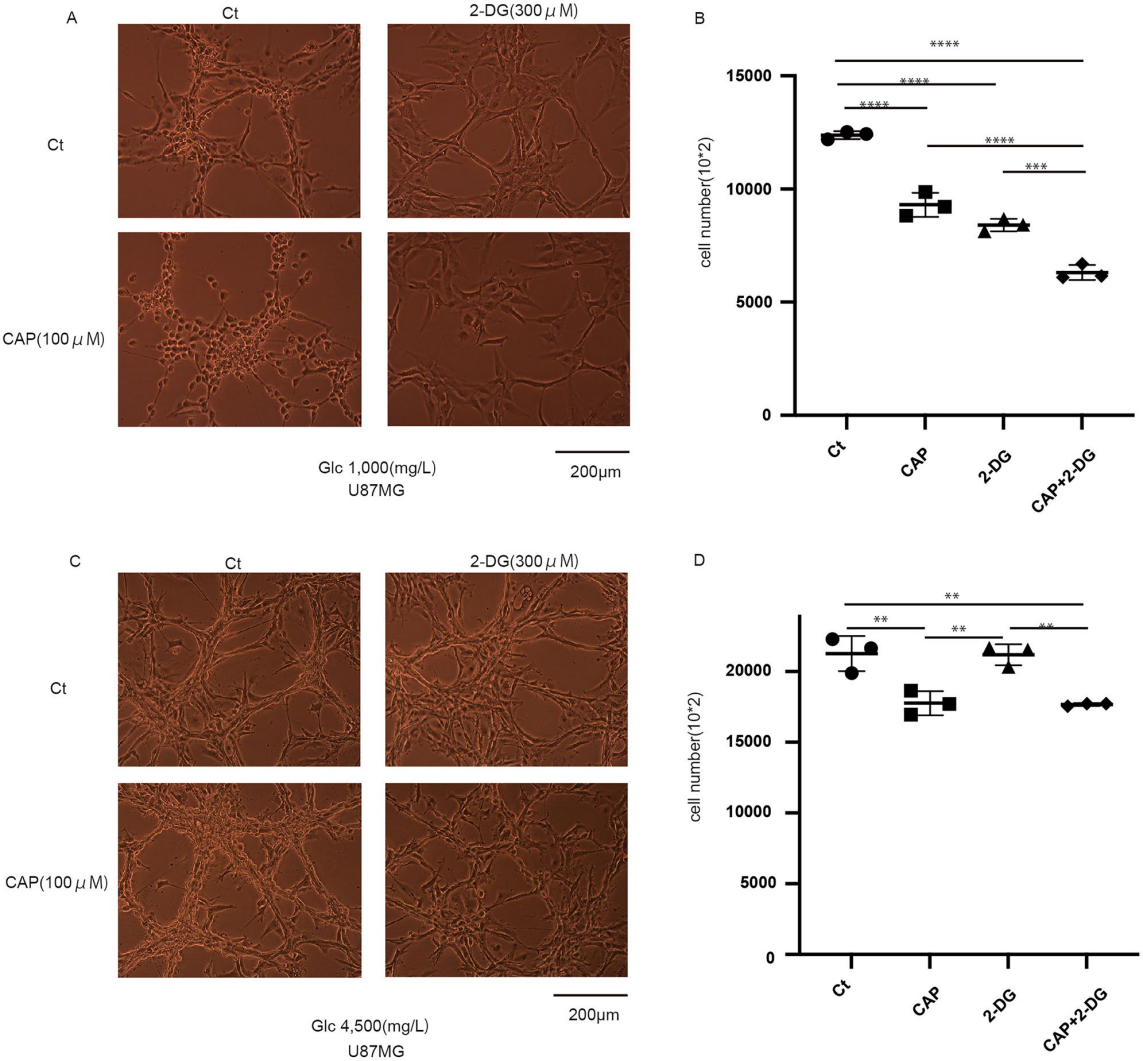
Effects of combined treatment, including chloramphenicol (CAP) and 2-deoxy-d-glucose (2-DG), under different glucose conditions in hypoxia. (**a**) The effect of the 5-day administration of CAP and 2-DG in U87 under normal glucose conditions for in hypoxia (O_2_ = 1%). The combined treatment was effective. (**b**) Cell number with each agent under a glucose concentration of 1000 mg/L (cells were seeded in a six-well plate and counted using a Coulter counter). (**c**) The effect of the 5-day administration of CAP and 2-DG in U87 under high glucose conditions. (**d**) Cell number with each agent under a glucose concentration of 4500 mg/L (cells were seeded in a six-well plate and counted using a Coulter counter). Values are presented as mean ± standard deviation. Statistical significance was assessed using the ordinary one-way analysis of variance test with Tukey’s multiple comparison test assessing Ct vs. CAP vs. 2-DG vs. CAP + 2-DG. **P < 0.01, ***P < 0.001, ****P < 0.0001.

In the biological environment, cells exist in hypoxic and low-nutrient conditions^14,15^. We assessed whether the effectiveness of combined treatment under hypoxic conditions (O_2_ = 1% is sufficient for triggering a hypoxic response (Supplementary Fig. S3a,b) and found that cell growth was inhibited under normal glucose conditions (Fig. 4a,b). Moreover, the effects of CAP and 2-DG were positive effect; however, under high glucose concentration, their effectiveness was decreased (Fig. 4c,d).

### Combined treatment is effective when delivered within patient-derived neurosphere cells

**Figure 5:**
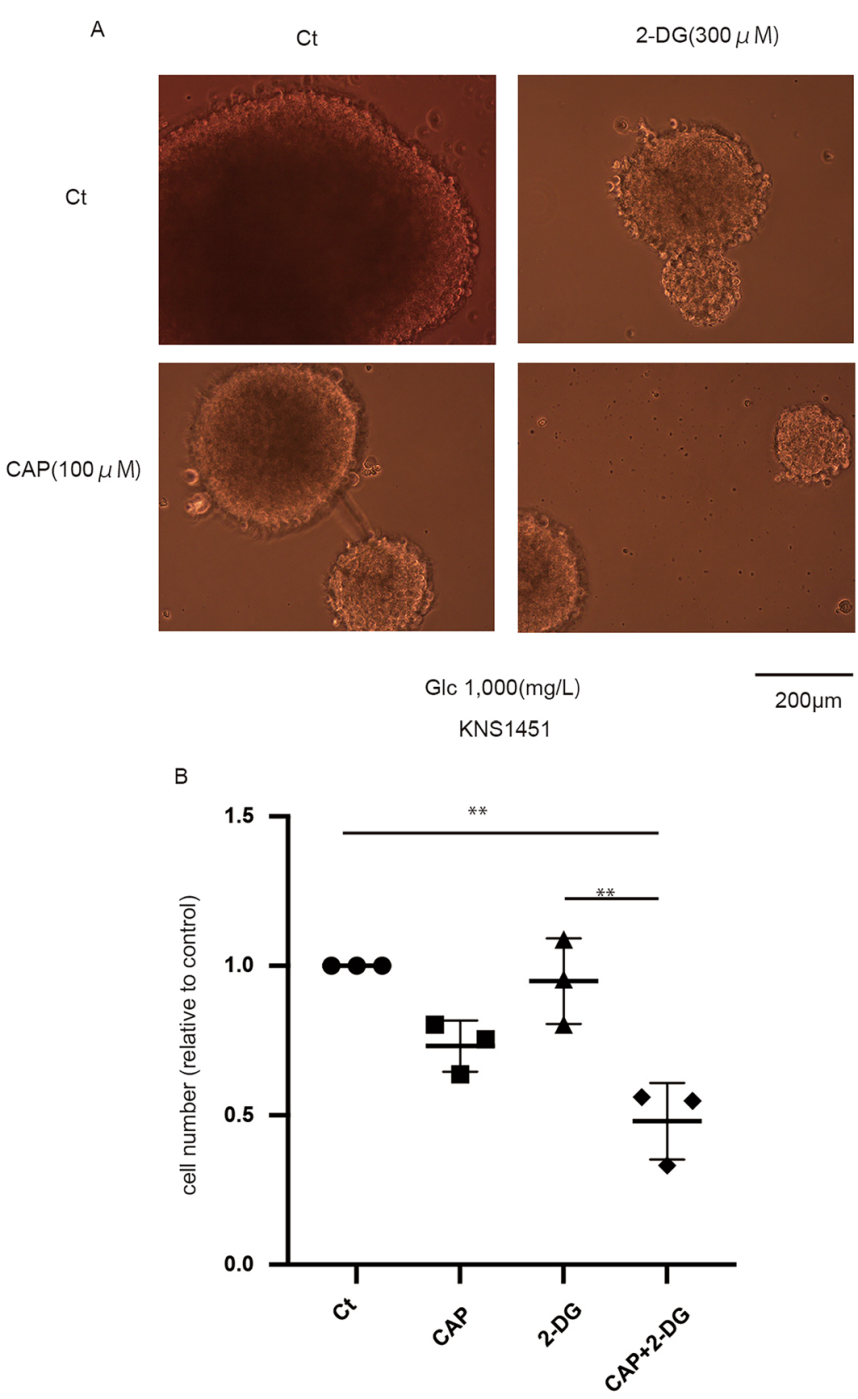
Effects of combined treatment in patient-derived stem-like cells. (**a**) The effect of the 7-day administration of chloramphenicol (CAP) and 2-deoxy-d-glucose (2-DG) in KNS1451 under normal glucose conditions. The combined treatment was effective. (**b**) Cell number with each agent under a glucose concentration of 1000 mg/L (cells were seeded in a six-well plate and counted using trypan blue). Values are presented as mean ± standard deviation. Statistical significance was assessed using the ordinary one-way analysis of variance test with Tukey’s multiple comparison test assessing Ct vs. CAP vs. 2-DG vs. CAP + 2-DG. **P < 0.01.

Finally, we used patient-derived stem-like cells to validate the effects of treatment mimicking an in vivo condition. Although single administration of CAP or 2-DG was ineffective, combined treatment was effective in neurosphere cells (Fig. 5a,b). The neurospheres were smaller, and the number of spheres decreased (Supplementary Fig. S4a,b).

### Combined treatment induces changes in iron dynamics

**Figure 6:**
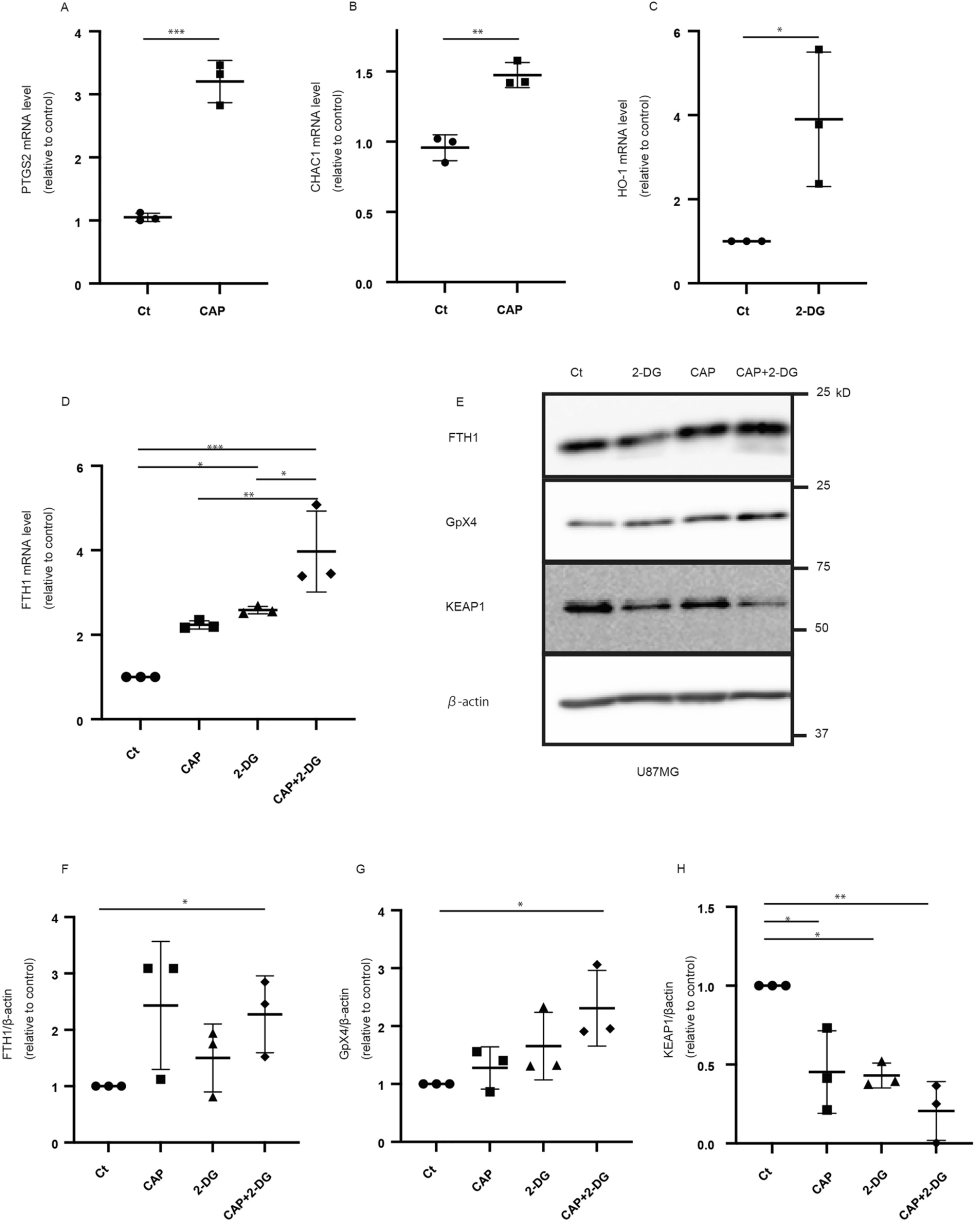
Iron dynamics were changed drastically by both agents. A change in ferroptosis was observed after the 5-day administration of chloramphenicol (CAP) and 2-deoxy-d-glucose (2-DG) treatment in U87. (**a**,**b**) Quantification of PTGS2 and CHAC1 mRNA expression (N = 3). (**c**) Quantification of HO-1 mRNA expression (N = 3). (**d**) Quantification of FTH1 mRNA expression (N = 3). (**e**) Western blot reveals that FTH1 and GpX4 levels increased in both agents, whereas KEAP1 levels decreased in both agents (N = 3). (**f**–**h**) Quantification results. Values are presented as mean ± standard deviation. Student’s *t*-test (**a**–**c**,**f**) or ordinary one-way analysis of variance with Tukey’s multiple comparison test (**d**,**g**,**h**) was performed to assess Ct vs. CAP vs. 2-DG vs. CAP + 2-DG. *P < 0.05, **P < 0.01, ***P < 0.001.

We investigated the mechanisms underlying the effectiveness of combined treatment. As previously reported, CAP injection results in cell death via ferroptosis under glucose-starved conditions^9^. Thus, we hypothesized that ferroptosis underlies the effectiveness of combined treatment. During CAP administration, the mRNA level of ferroptosis markers, including PTGS2 and CHAC1, increased (Fig. 6a,b, Supplementary Fig. S5a). Regarding the mRNA of HO-1 levels, one of the markers related to iron dynamics also increased in 2-DG administration (Fig. 6c). The mRNA levels of FTH1 increase upon treatment with either agent. However, the highest level of FTH1 mRNA was observed upon combined treatment, demonstrating the combination effect of these two agents (Fig. 6d, Supplementary Fig. S5b). In addition, the expression of ferroptosis-related proteins, including FTH1, GpX4, and KEAP1, demonstrated a combination effect (Fig. 6e–h). In particular, the expression of FTH1 and GpX4 increased using CAP and 2-DG. However, the expression of KEAP1 decreased using both CAP and 2-DG. These results imply that the two agents, CAP and 2-DG, cause drastic iron dynamic changes and that ferroptosis is one of the pathways that result in cell death. To exclude the possibility of other types of cell death, including apoptosis, we performed immunoblotting analysis to assess the expression of caspase-3, which implies apoptosis (Supplementary Fig. S6a,b). Furthermore, combined treatment with 2-DG and other ferroptosis inducers, namely sodium selenite, was also effective (Supplementary Fig. S7a,b)^9,20^.

### Cell death is inhibited by deferoxamine

**Figure 7:**
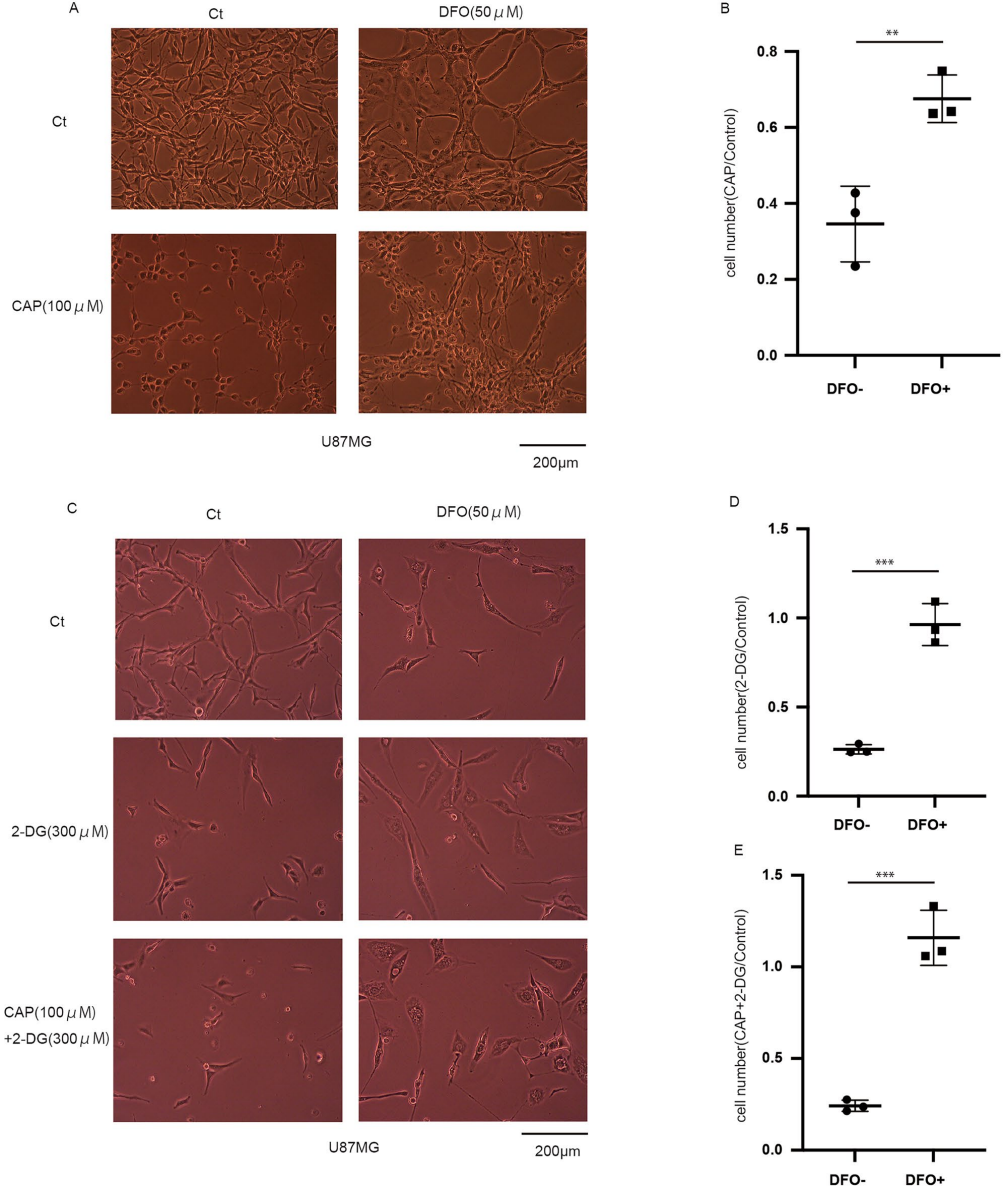
Inhibition of ferroptosis by deferoxamine (DFO). (**a**,**b**) In U87, cell number assay reveals that chloramphenicol (CAP) and DFO (50 μM, added 72 h after CAP injection) reduced cell death after 5 days. Values are presented as mean ± standard deviation. (N = 3). (**c**–**e**) In U87, the cell number assay revealed that 2-deoxy-d-glucose (2-DG) or CAP + 2-DG and DFO reduced cell death after 3 days, wherein DFO (50 μM) was added initially. Values are presented as mean ± standard deviation. (N = 3). A Student’s *t*-test was performed. **P < 0.01, ***P < 0.001.

Considering the results of mRNA and protein expression, CAP and 2-DG may induce ferroptosis, emphasizing the effectiveness of combined treatment. To prove this, we used DFO, an iron chelator and inhibitor of ferroptosis. DFO inhibited the effects of CAP (Fig. 7a,b) and those of 2-DG and CAP with 2-DG (Fig. 7c–e). Considering these results, cell death caused by CAP and 2-DG was inhibited by DFO, and one of the mechanisms of these agents is ferroptosis.

## Discussion

Little is known about the relationship between glioblastoma and the mitochondria. We focused on the mitochondria because they are key organelle targets in treating glioblastoma^21^. In addition, hypoglycemic and hypoxic conditions exist in the tumor environment^14,15^. In our previous study, we focused on the development of glioblastoma treatment under hypoglycemic conditions^9^. The findings of this prior study were as follows: mitochondria were activated under glucose-starved conditions, and antimicrobial agents were effective under glucose-starved conditions. Because the tumor environment is hypoglycemic and deficient in various nutrients, previous findings represented promising treatments. However, under glucose-starved conditions, the glucose level (100 mg/L) was much lower than under normal glucose conditions (1000 mg/L)^9^. Considering tumor heterogeneity, some high-nutrient lesions may have higher glucose levels than low-nutrient lesions. It is important to develop an effective treatment with various oxygen concentrations in normal and low glucose conditions to overcome these malignant tumors. Therefore, in this study, we focused on normal glucose and hypoxic conditions and developed a combined treatment using CAP and 2-DG. This treatment inhibited cell growth drastically under normal glucose conditions in both normal and hypoxic conditions. Thus, combined treatment can be promising for glioblastoma patients.

According to our results, combined treatment was ineffective under high glucose (4500 mg/L) compared to normal glucose conditions, highlighting the importance of maintaining glucose levels within the normal range. According to a previous study, blood glucose levels are associated with prognosis in patients with glioblastoma, with higher glucose levels resulting in poorer prognoses^22^. In addition, the OCR was higher under normal glucose than under high glucose conditions, suggesting that mitochondria-targeted therapy is more effective under normal glucose conditions than under high glucose conditions^9^, with a glucose concentration of 1000 mg/L being considered normal in humans.

The blood–brain barrier (BBB) blocks some agents from invading the brain^23^. Thus, whether an agent can cross the BBB should be considered. Recently, nanotherapeutic techniques have been developed to overcome BBB to enhance glioblastoma treatment^23^. From various agents available, we selected two agents, CAP and 2-DG, that can easily cross the BBB and directly affect the mitochondria^24,25^.

CAP is an antimicrobial agent that induces mitochondrial dysfunction^26^. Moreover, this agent has been used to treat meningitis^27^. Dunkle et al.^27^ reported that the effective blood concentration for premature newborns with central nervous system infection was 46–154 μM. Furthermore, a 100 μM concentration is reasonable and less harmful. In addition, when using this agent, drug repositioning is expected. Metformin has recently been used to treat cancers, including glioblastomas^28^. Kim et al.^28^ reported that 2-DG combined with metformin inhibits glioblastoma cell proliferation. We investigated the effect of metformin under glucose-starved conditions, and our study reported that CAP was effective under glucose-starved conditions^9^, whereas metformin was not very effective (Supplementary Fig. S8). This difference is probably due to differences in the mechanisms of action of these two drugs. CAP inhibits mitochondrial ribosomes and causes mitochondrial dysfunction^26^. Metformin reportedly inhibits the mitochondrial complex 1; however, another theory has been proposed, and the exact mechanism has not been fully elucidated^29^. CAP is effective under both glucose-starved and normal conditions. Therefore, we believe that CAP is a key drug for treating glioblastoma.

2-DG is used to treat cancers targeting glycolysis^12^. Stein and Raez conducted a clinical trial to treat cancers or solid tumors using 2-DG and presented its safety at appropriate concentrations. Stein recommended a 45 mg/kg dose, whereas Raez recommended a safe dose of 63 mg/kg/day^12,13^. In addition, the C_max_ of a 45 mg/kg dose is 73.7 μg/ml (449 μM), whereas that of a 63 mg/kg/day dosage is 116 μg/ml (almost 700 μM). However, Sasaki et al.^30^ reported that although 2-DG successfully treats cancer, effective doses induce serious adverse effects. They, therefore, recommended a new device that delivers 2-DG in poly-lactic-co-glycolic acid nanoparticles. In our study, the concentration of 2-DG was 300 μM, which is within the safe range.

2-DG inhibits glycolysis and provides conditions similar to glucose starvation. In glucose-starved conditions, as previously reported, mitochondria become dominant. 2-DG also increases the expression of COX1 and OXPHOS, implying mitochondrial dominance. Additionally, we consider that this nutrient deprivation leads to increased fatty acid oxidation activity and suppose the OXPHOS increase is due to fatty acid activations. Etomoxir is used to detect the activity of fatty acid oxidation, and it is reported that high dose etomoxir inhibits mitochondrial complex 1^31^. OXPHOS decreased after the etomoxir injection, although the decrease was less than expected. Thus, there may be other pathways, including the amino acid pathway. Further investigation is needed to elucidate this.

Based on the study results, ferroptosis may have occurred. Several markers of ferroptosis, including KEAP1, NRF2, HO-1, GpX4, TFRC, FTH1, and xCT, have recently been reported^17^. A previous study has shown that CAP induces ferroptosis via the p-p62-KEAP1-NRF2-HO-1 pathway^9^. In this study, we used two different agents; it was, therefore, difficult to confirm the existence of a single ferroptosis stream. However, the two agents drastically affected iron dynamics and showed a combination effect on FTH1, GpX4, and KEAP1. Further, to confirm whether these agents induce ferroptosis, it is important to use an inhibitor of this pathway; we used DFO accordingly. DFO inhibited both agents, and we believe that the mechanism underlying cell death, in this case, is ferroptosis. In this study, we assessed other pathways of cell death, including apoptosis and necroptosis. The expression of caspase 3, which implies apoptosis, was not changed in each agent. Moreover, an increase in the level of RIP3K mRNA, which is related to necroptosis, was not detected. In addition, one reason 2-DG causes ferroptosis may be that glucose deprivation induces the blocking of the serine synthesis pathway, a pathway derived from glycolysis^32^. This leads to the depletion of glutathione, an activator of GpX4 protecting against ferroptosis^33^.

One limitation of this study is that data were collected in vitro rather than in vivo; thus, further in vivo studies on mice are required. However, the development of effective treatment is worth reporting. In conclusion, we developed an effective treatment combining CAP with 2-DG to treat glioblastoma cell lines under normal glucose conditions. Therefore, the results of our study seem beneficial for developing treatments for glioblastoma.

## Methods

### Cell culture

U87MG (U87) and U373 were obtained from the American Type Culture Collection (Manassas, VA, USA) (certified by BEX [Japan]). The use of cell lines for this study was approved by the Ethics Committee of the Graduate School of Medical Science, Kyushu University. Written consent was obtained from all patients.

The cells were cultured, as previously reported^9^. Similarly, an original patient-derived glioblastoma cell line, KNS1451, obtained from Kyushu University Brain Tumor Bank, was cultured as previously reported^9^. Genetic analysis revealed that KNS1451 harbored mutations, such as phosphatase and tensin homolog, tumor protein 53, neurofibromatosis 1, and telomerase reverse transcriptase promoter C250T. This cell line is classified as an aggressive mesenchymal type.

### Quantitative real-time PCR

Real-time PCR was performed, as previously described^9^. Ribosomal 18S rRNA was evaluated as an internal control. Primer sequences are shown in Supplementary Table S1.

### Immunoblotting analysis

Immunoblotting analysis was performed, as previously described^9^. The following antibodies were used in this study: mouse monoclonal anti-MTCO1 (#ab14705, Abcam, RRID:AB_2084810), mouse monoclonal anti-β-actin (#A5441, Sigma-Aldrich, RRID_2766243), rabbit polyclonal anti-GPX4 (#52455, CST, RRID:AB_2924984), rabbit monoclonal anti-KEAP1 (#8047, CST, RRID:AB_10860776), rabbit polyclonal anti-FTH1 (#3998, CST), rabbit polyclonal anti-Caspase-3 (#9662, CST), rabbit monoclonal anti-HIF-1α antibody (#14179, CST, RRID:AB_2622225), anti-rabbit IgG HRP-linked (#7074, CST), and anti-mouse IgG HRP-linked (#7076, CST). The membranes were cut prior to hybridization with antibodies.

### Cell number counts

The cells were seeded (1 × 10^5^ in a 6-well dish except for that in Fig. 1 in which 2 × 10^4^ cells were seeded) in triplicate and cultured in DMEM (containing glucose, CAP (034-10572, Wako), 2-DG (D8375, Sigma-Aldrich), DFO (205-314-3, Sigma-Aldrich), SS (10102-18-8, WAKO), and metformin (1115-70-4, Tokyo Chemical Industry) at each concentration. This culture medium was replaced every 3 days; the cells were trypsinized and counted using a Coulter counter (Beckman Coulter, USA) or TC 20 automated cell counter (BIO-RAD, #1450101J1, USA) with trypan blue. To assess the proliferation of stem-like cells, the cells (5 × 10^4^ in a 6-well dish) were seeded in triplicate or more and cultured in DMEM/Ham’s F12 (containing CAP and 2-DG at each concentration) for 7 days. On the 7th day, the sphere number and size were evaluated. After centrifugation at 3000×*g* for 2 min, the cell pellets were trypsinized, added to the medium, and counted using the TC 20-cell automated cell counter with trypan blue.

### Hypoxic experiments

Hypoxic conditions were achieved using a personal CO_2_ multi-gas incubator (ASTEC) with 1% O_2_ and 5% CO_2_. The O_2_ concentration was automatically checked by a Gas Cylinder Auto Changer (model 8420, Waken).

### Seahorse XF24 flux analyzer

Mitochondrial OXPHOS and glycolytic activity can be measured using the OCR methods with an XFe24 analyzer (Seahorse Biosciences, USA). Basal OCR was measured using the Seahorse XF24 Flux analyzer, as previously described^9^. In addition to this, fatty acid activity can be measured using 40 μM etomoxir (#1905, sigma-aldrich) and 0.5 mM l-carnitine (#541-15-1, TCI)^18,19^. Seahorse XF24 microplates were seeded with 1 × 10^5^ cells/well (before seeding, cells were incubated for 2 days under each condition) and incubated at 37 °C for approximately 16 h. After analysis, the cells were seeded with a similar number of cells in another 96-well plate, trypsinized, and counted at the start of the analysis. The results were normalized to the number of cells.

### Statistical analyses

Statistical analyses are described in the figure legends. Data are presented as mean ± standard deviation. Significant differences between groups were examined using a one-way analysis of variance or Student’s *t*-test with GraphPad Prism version 9 (GraphPad Prism Software Inc.). All experiments were repeated at least thrice.

## Supplementary Information

Supplementary Information.

## Data Availability

All data generated or analyzed during this study are included.
