# Supplementary material for: Induction of glioblastoma cell ferroptosis using combined treatment with chloramphenicol and 2-deoxy-d-glucose
Source: Sci Rep. 2023 Jun 28;13:10497. doi: 10.1038/s41598-023-37483-5 (PMC10307808; doi:10.1038/s41598-023-37483-5)

Induction of glioblastoma cell ferroptosis using combined treatment with chloramphenicol and 2-deoxy-D-glucose

**Kenji Miki, Mikako Yagi, Naoki Noguchi, Yura Do, Ryosuke Otsuji, Daisuke Kuga, Dongchon Kang, Koji Yoshimoto, and Takeshi Uchiumi**

**Supplementary Legends**

**Supplementary Figure S1. Effects of combined treatment, including chloramphenicol (CAP) and 2-deoxy-D-glucose (2-DG), under a normal glucose condition for 3 days.** (**a**) The effect of the 3-day administration of CAP and 2-DG in U87 in normal glucose condition; the combined treatment is effective. (**b**) Cell number with each agent under a glucose concentration of 1000 mg/L (cells were seeded in a six-well plate and counted using a Coulter counter). (**c**) The effect of the 3-day administration of CAP and 2-DG in U373 in normal glucose condition; the combined treatment is effective. (**d**) Cell number with each agent under a glucose concentration of 1000 mg/L (cells were seeded in a six-well plate and counted using a Coulter counter). Values are presented as mean ± standard deviation. Statistical significance was assessed using the ordinary one-way analysis of variance test with Tukey’s multiple comparison test assessing Ct vs. CAP vs. 2-DG vs. CAP + 2-DG. ^***^P < 0.001, ^****^P < 0.0001.

**Supplementary Figure S2. Etomoxir decreases the effect of 3-day administration of 2-DG for increased oxygen consumption rate (OCR).** (**a**) Traces of OCR in control and 2-DG and with or without etomoxir administration (30 μM) and (**b**) quantification of maximal respiration. Values are presented as mean ± standard deviation. Student’s *t*-test was performed 2-DG vs. 2-DG+Etomoxir. ^*^P < 0.05.

**Supplementary Figure S3. Hypoxia is sufficient for triggering a hypoxic response. (a)** The expression of HIF-1 alpha in normal and hypoxic conditions and (**b**) quantification. Values are presented as mean ± standard deviation. Student’s *t*-test was performed 02=21% vs. O2=1%. ^*^P < 0.05.

**Supplementary Figure S4. Effects of combined treatment in patient-derived stem-like cells.** The effect of the 7-day administration of chloramphenicol (CAP) and 2-deoxy-D-glucose (2-DG) in KNS1451 under normal glucose conditions; the combined treatment was effective. (**a**) Sphere number (**b**) sphere size of 20 fields of view in each agent under a glucose concentration of 1000 mg/L. Values are presented as mean ± standard deviation. Statistical significance was assessed using the ordinary one-way analysis of variance test with Tukey’s multiple comparison test assessing Ct vs. CAP vs. 2-DG vs. CAP + 2-DG. ^*^P < 0.05, ^**^P < 0.01, ^***^P < 0.001, ^****^P < 0.0001.

**Supplementary Figure S5. Iron dynamics are affected by both agents for 3 days.** The effect of the 3-day administration of CAP and 2-DG in U87 under normal glucose conditions. A change in the ferroptosis marker, including CHAC1 (**a**) and FTH1 (**b**), is shown. The combination effect of the two agents can also be seen. Values are presented as mean ± standard deviation. Ordinary one-way analysis of variance with Tukey’s multiple comparison tests was performed assessing Ct vs. CAP vs. 2-DG vs. CAP + 2-DG. ^*^P < 0.05, ^**^P < 0.01, ^***^P < 0.001, ^****^P < 0.0001.

**Supplementary Figure S6. Apoptosis does not occur in CAP and DG treatment for 5 days.**

**(a)** In U87, western blot reveals that caspase-3 levels in each agent (N = 3). (**b**) Quantification results. Values are presented as mean ± standard deviation. Ordinary one-way analysis of variance with Tukey’s multiple comparisons test was performed assessing Ct vs. CAP vs. 2-DG vs. CAP + 2-DG.

**Supplementary Figure S7. Effects of combined treatment, including 2-deoxy-D-glucose (2-DG) and sodium selenite (SS).**

(**a**) The effect of the 3-day administration of SS and 2-DG in U87 in normal glucose conditions. The combined treatment is also effective. (**b**) Cell number with each agent under a glucose concentration of 1000 mg/L (cells were seeded in a 6-well plate and counted using a Coulter counter). Values are presented as mean ± standard deviation. Statistical significance was assessed using the ordinary one-way analysis of variance test with Tukey’s multiple comparison test assessing Ct vs. 2-DG vs. SS vs. 2-DG+SS. ^**^P < 0.01, ^****^P < 0.0001.

**Supplementary Figure S8. Effects of metformin under glucose-starved conditions**

(**a**) The effect of metformin in U87 (5 × 10^4^ cells were seeded in a 12-well dish) in glucose-starved condition (100 mg/L) for 3-day administration. (**b**) Cell viability with each concentration of metformin (cells were seeded in a 12-well plate and counted using a Coulter counter). Values are presented as mean ± standard deviation. Statistical significance was assessed using the ordinary one-way analysis of variance test with Tukey’s multiple comparisons test assessing Ct vs. 10 μM vs. 1000 μM. ^*^P < 0.05.

**Supplementary Table S1.** Primer sequences

| Target | Forward (5'→3') | Reverse (3'→5') |
| --- | --- | --- |
| 18S | AAACGGCTACCACATCCAAG | CCTCCAATGGATCCTCGTTA |
| PTGS2 | TGAGCATCTACGGTTTGCTG | TGCTTGTCTGGAACAACTGC |
| FTH1 | TGACAAAAATGACCCCCATT | CAGGGTGTGCTTGTCAAAGA |
| HO-1 | GGC CTC CCTGTACCACATCT | AGACAGGTCACCCAGGTAGC |
| CHAC1 | AGATCATGAGGGCTGCACTT | CCAGACGCAGCAAGTATTCA |
| RIP3K | CTTCCAGGAATGCCTACCAA | TCCATTTCTGTCCCTCCTTG |

**Supplementary Figure S1**

**
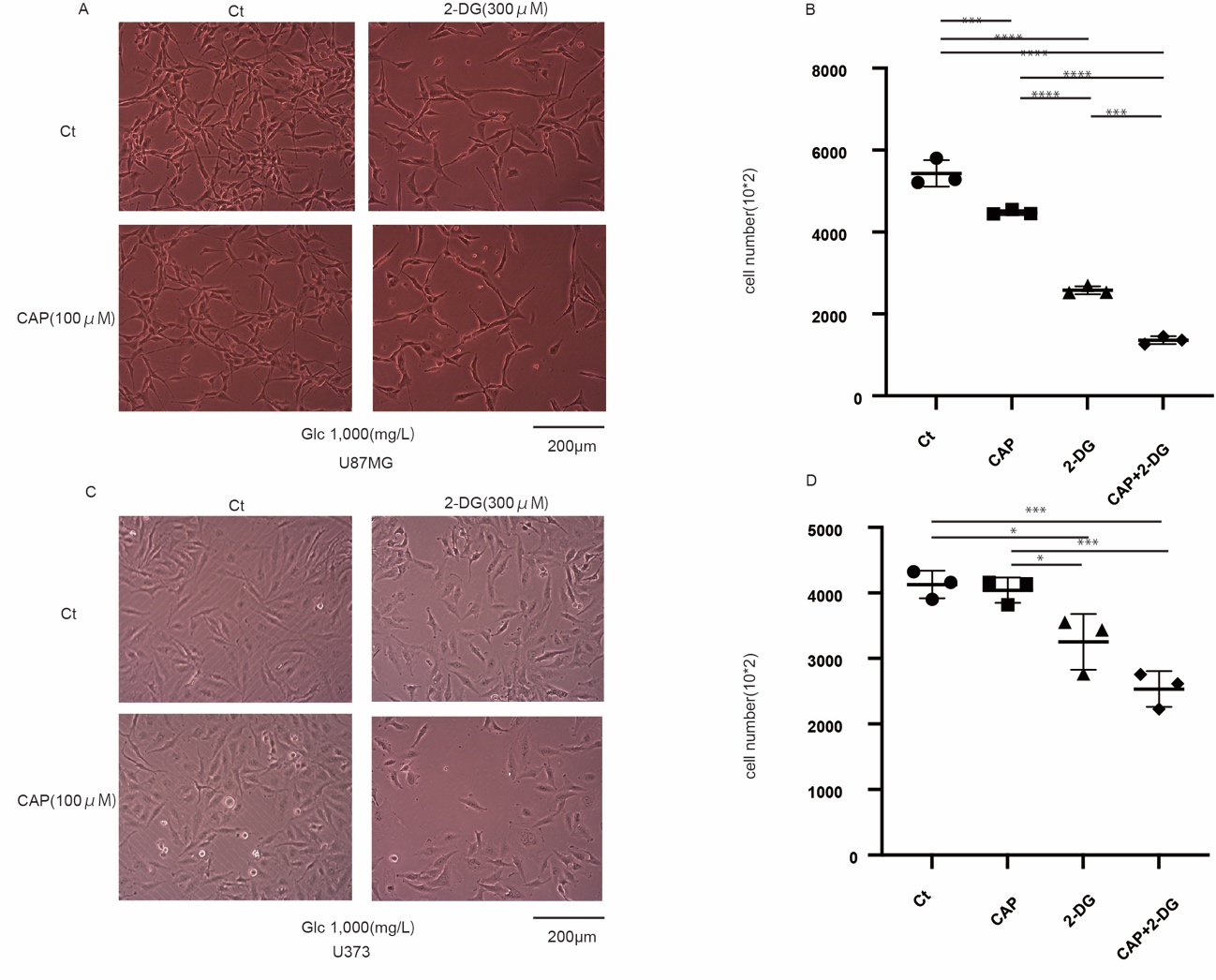
**

**Supplementary Figure S2**


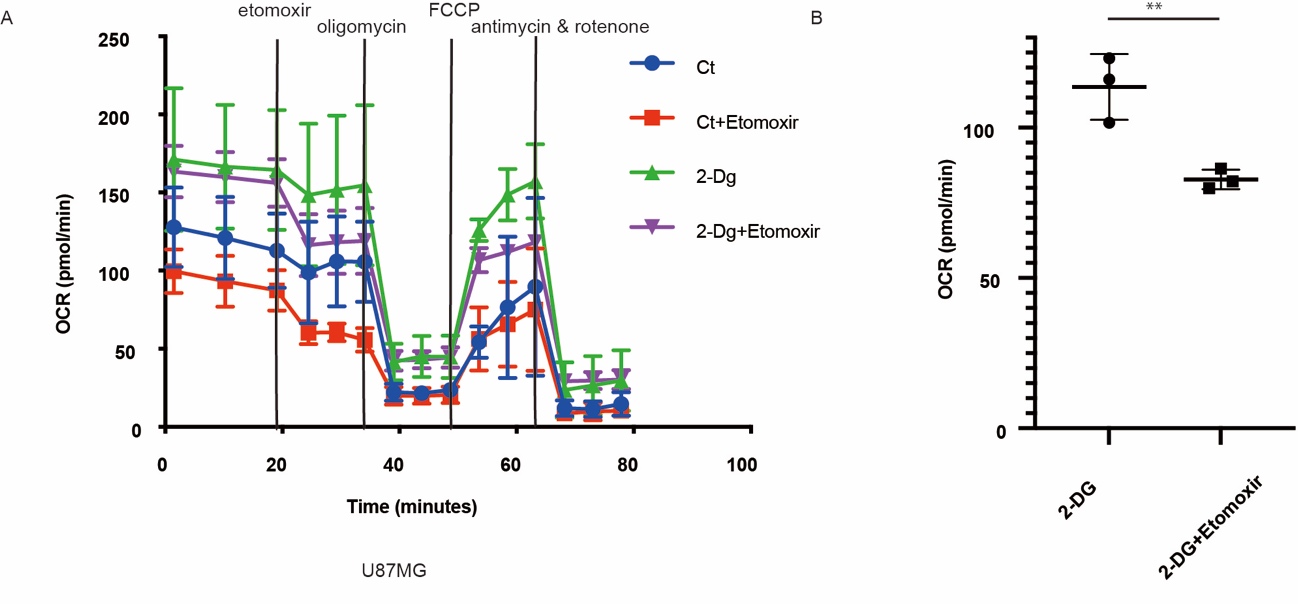


**Supplementary Figure S3**


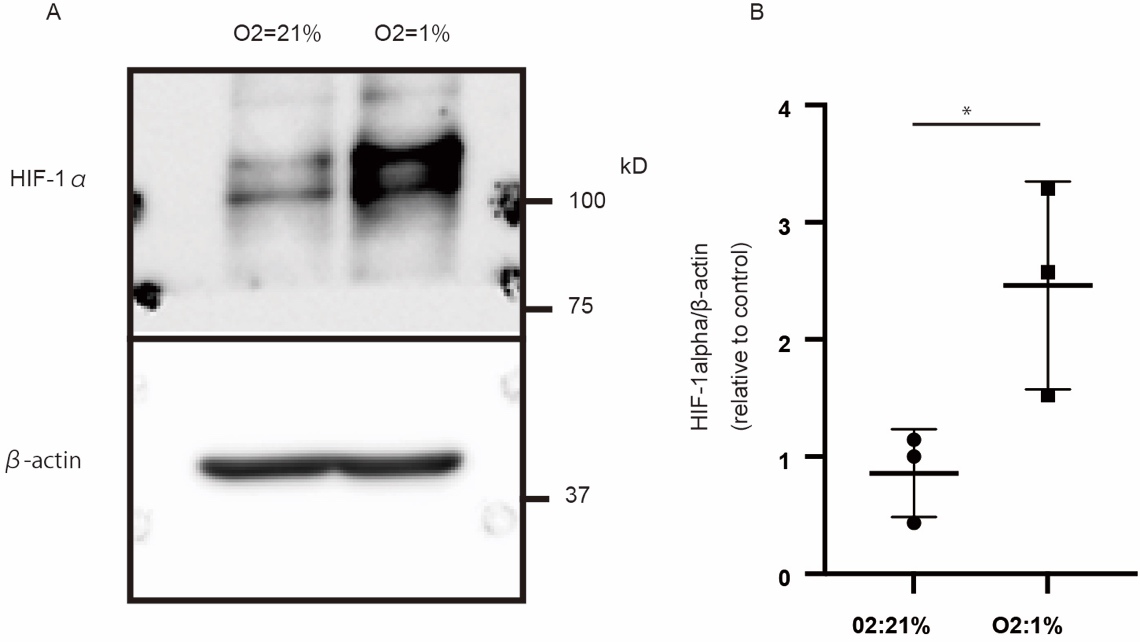


**Supplementary Figure S4**

**
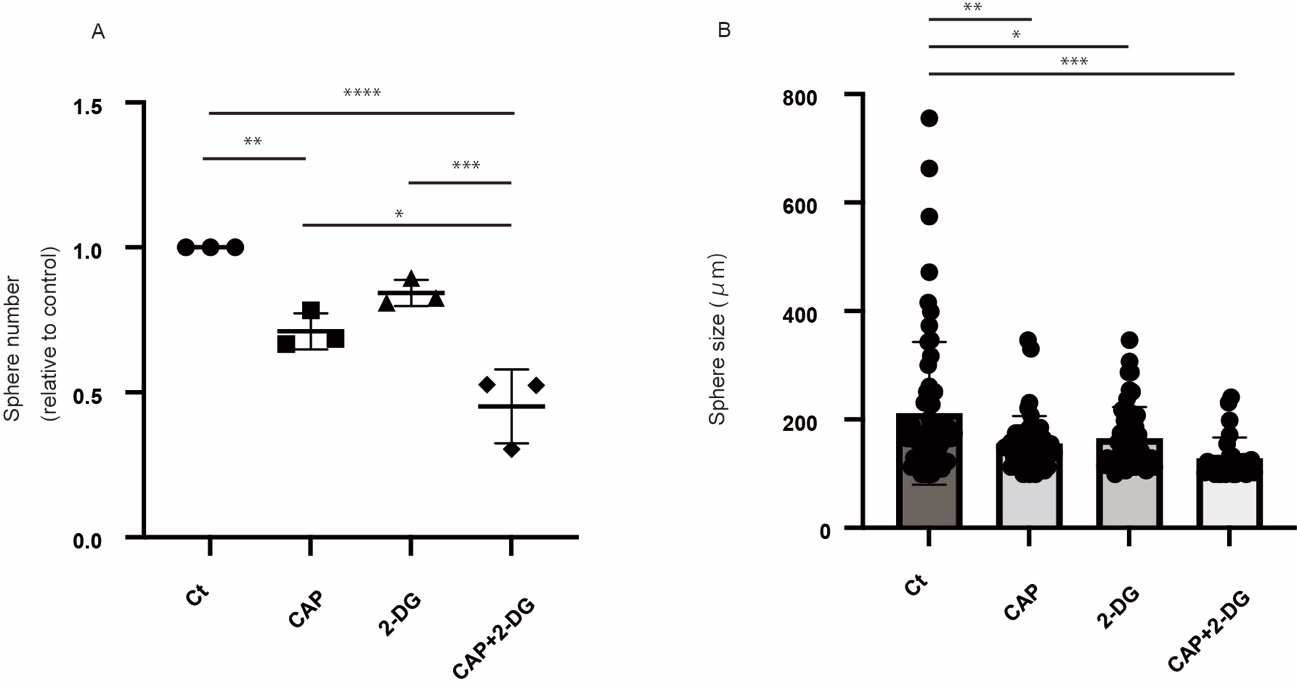
**

**Supplementary Figure S5**

**
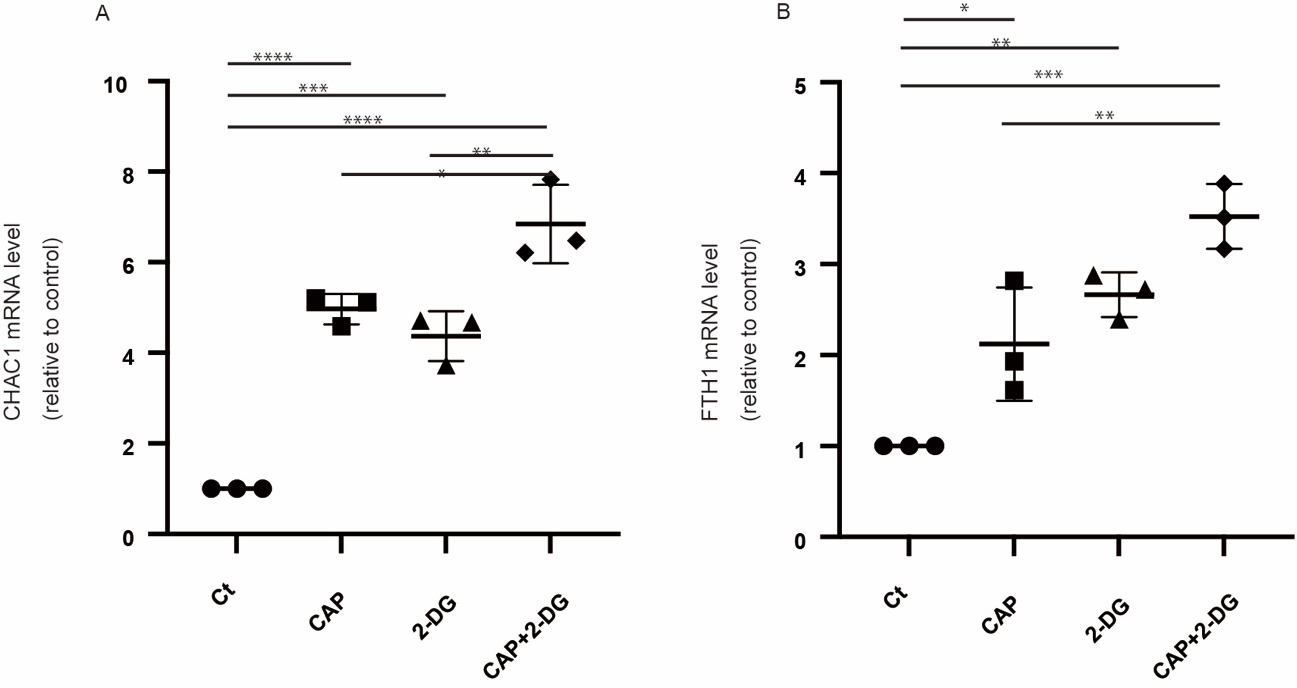
**

**Supplementary Figure S6**

**
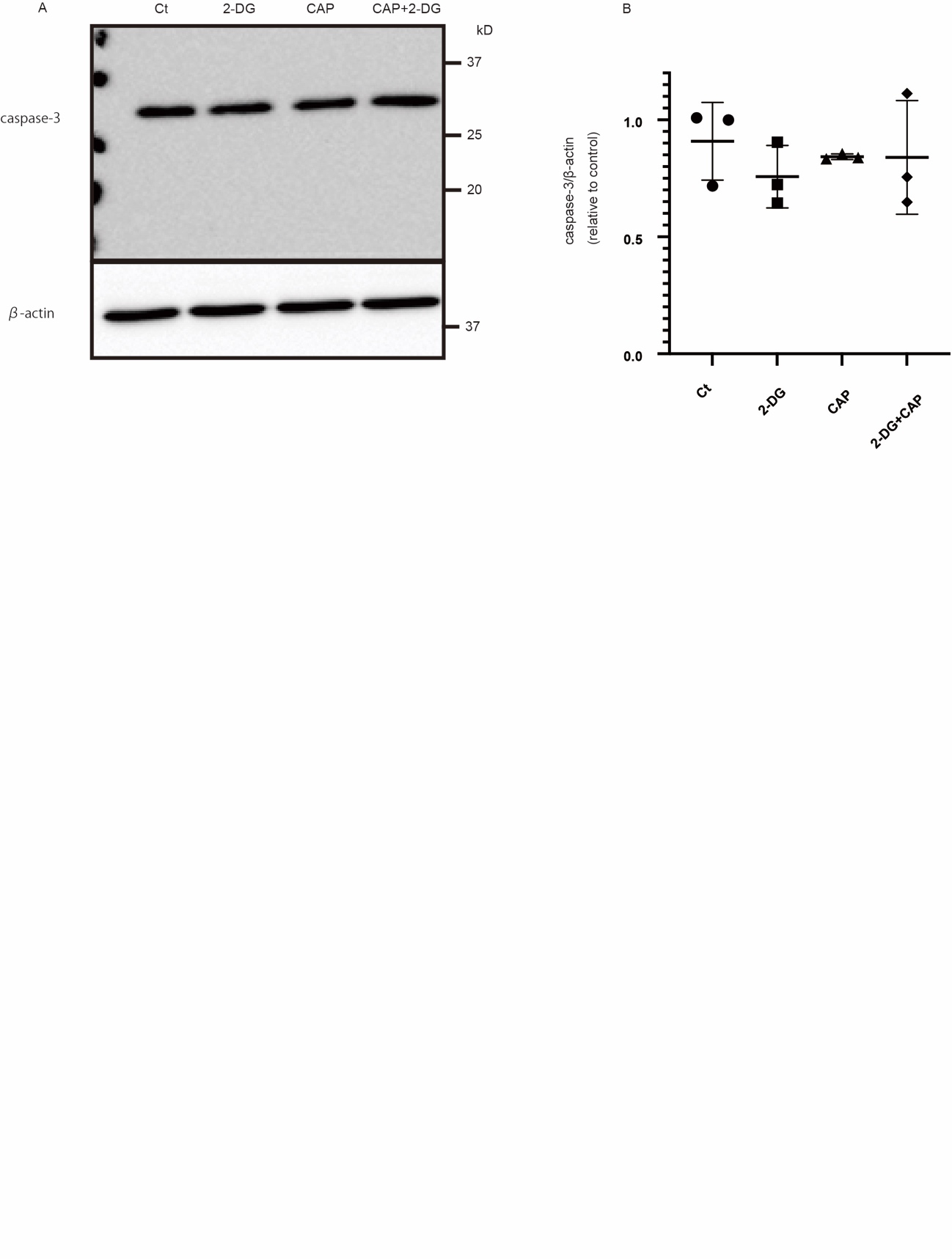
**

**Supplementary Figure S7**

**
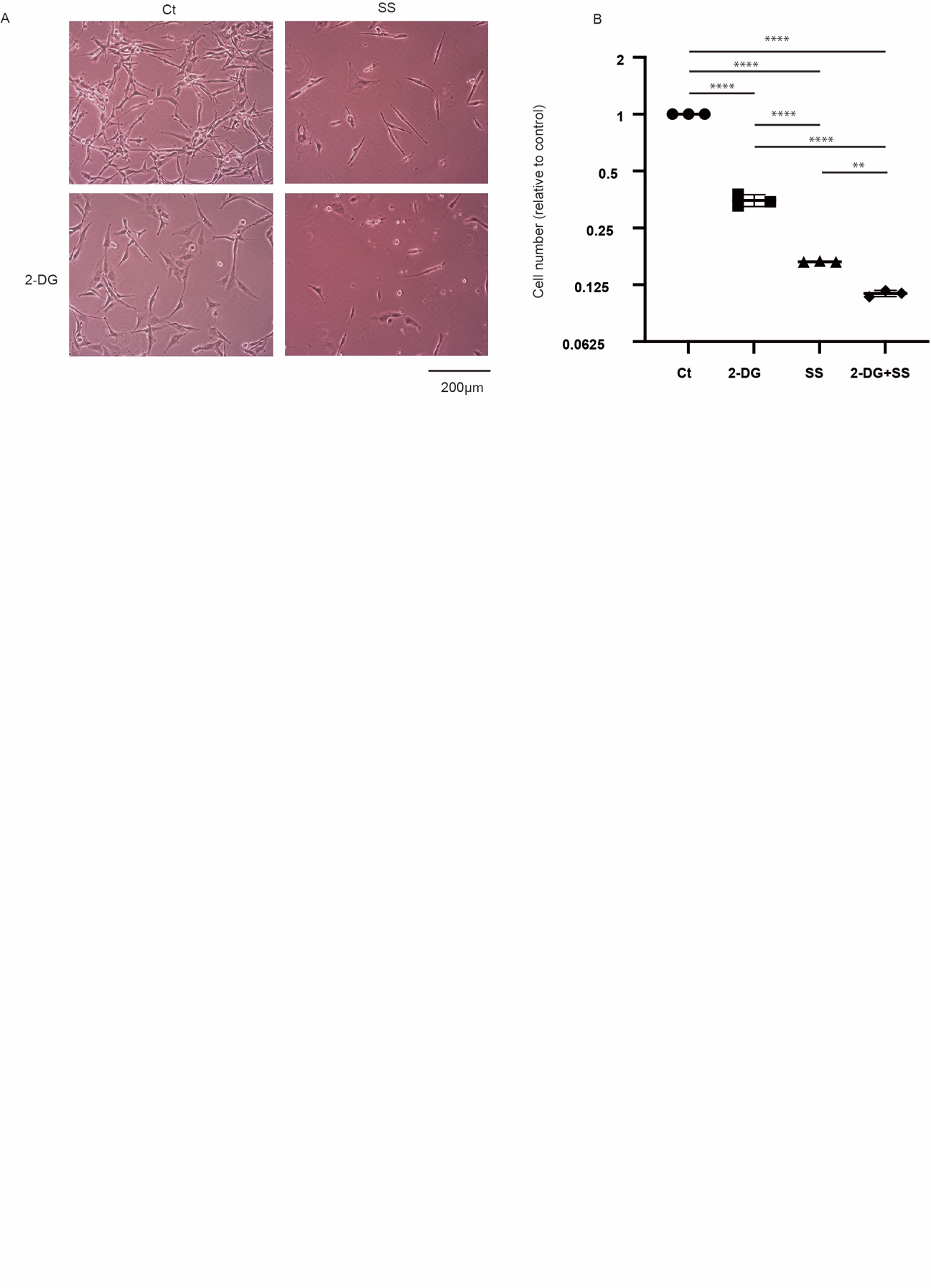
**

**Supplementary Figure S8**


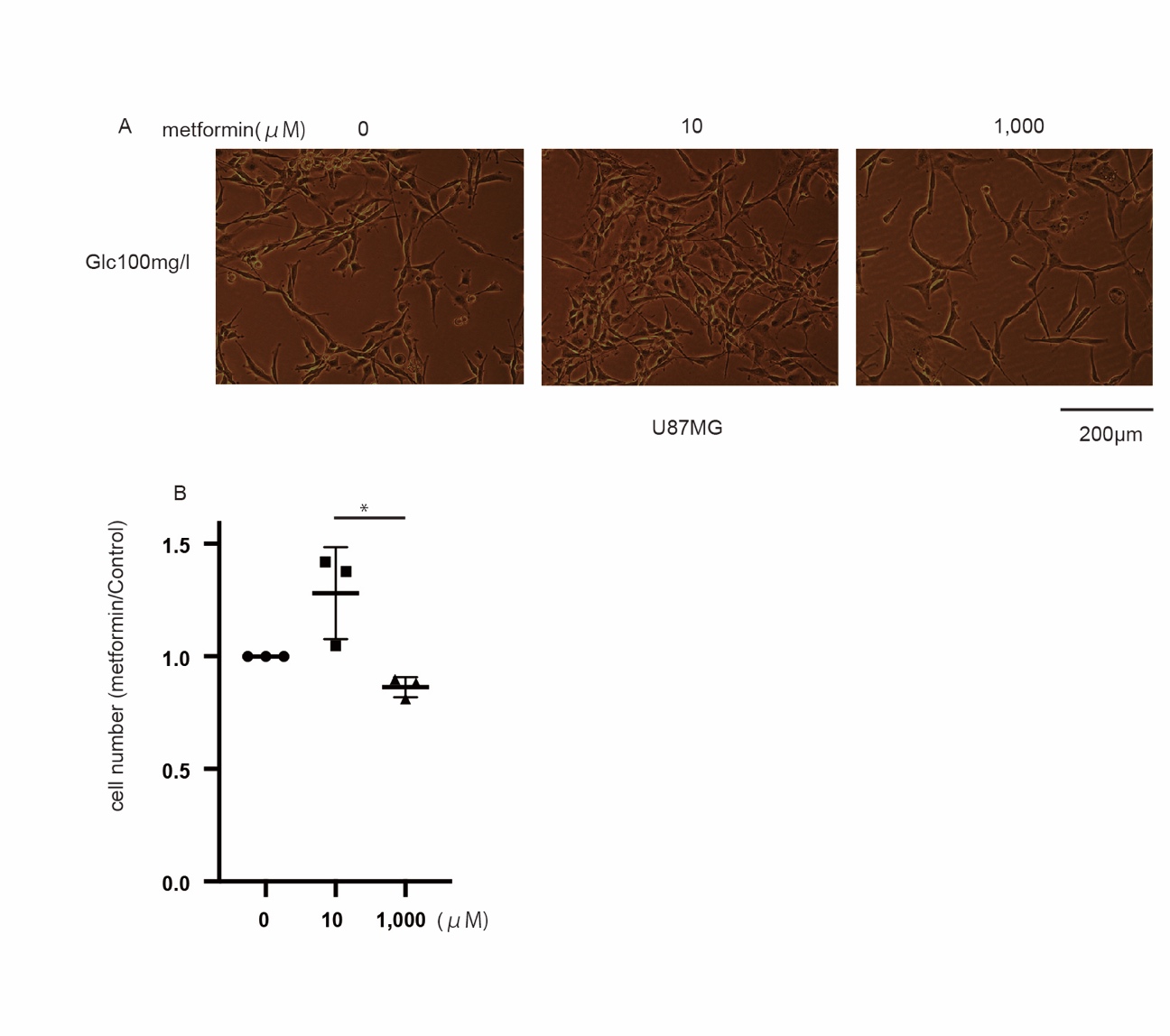


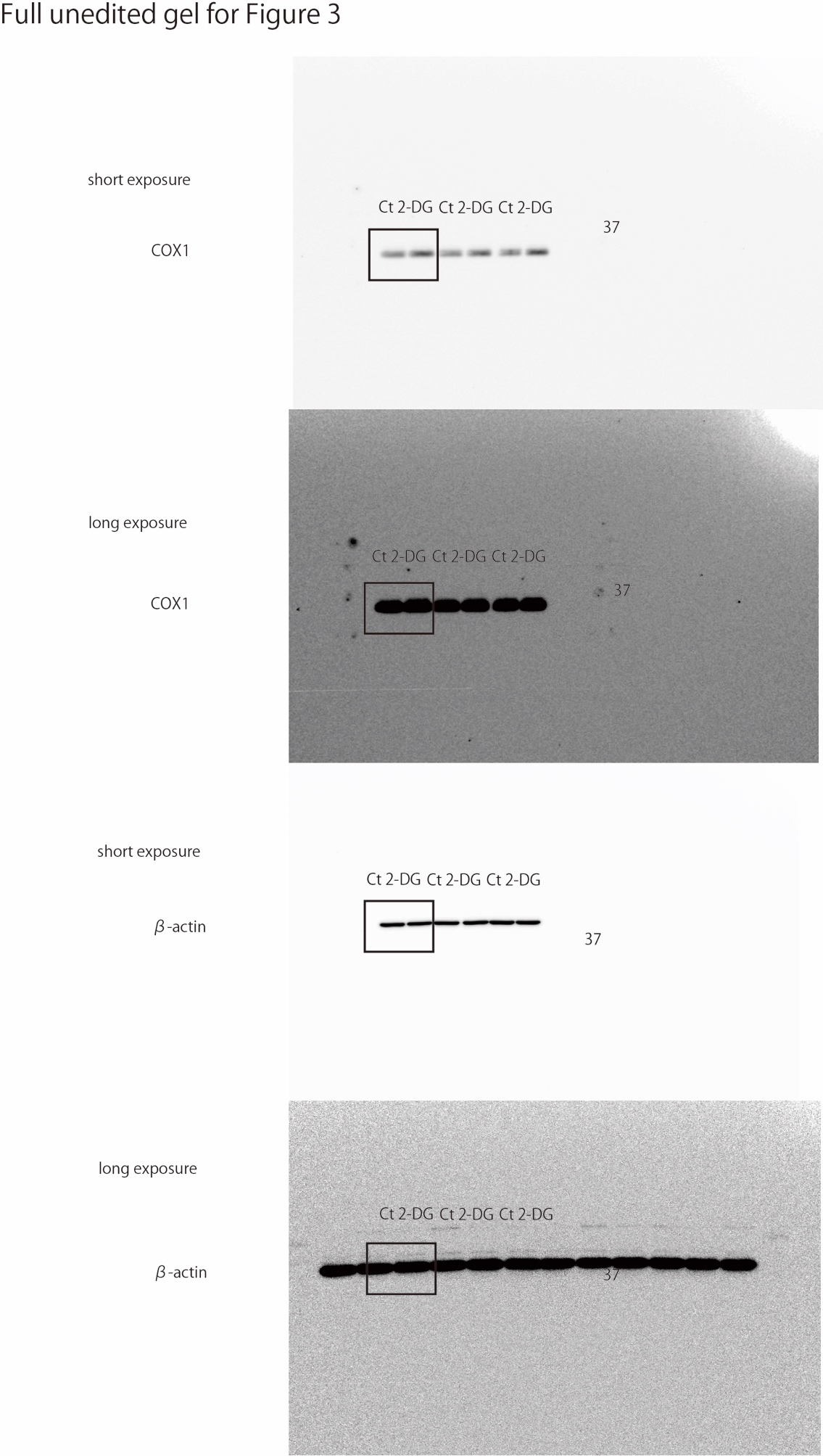


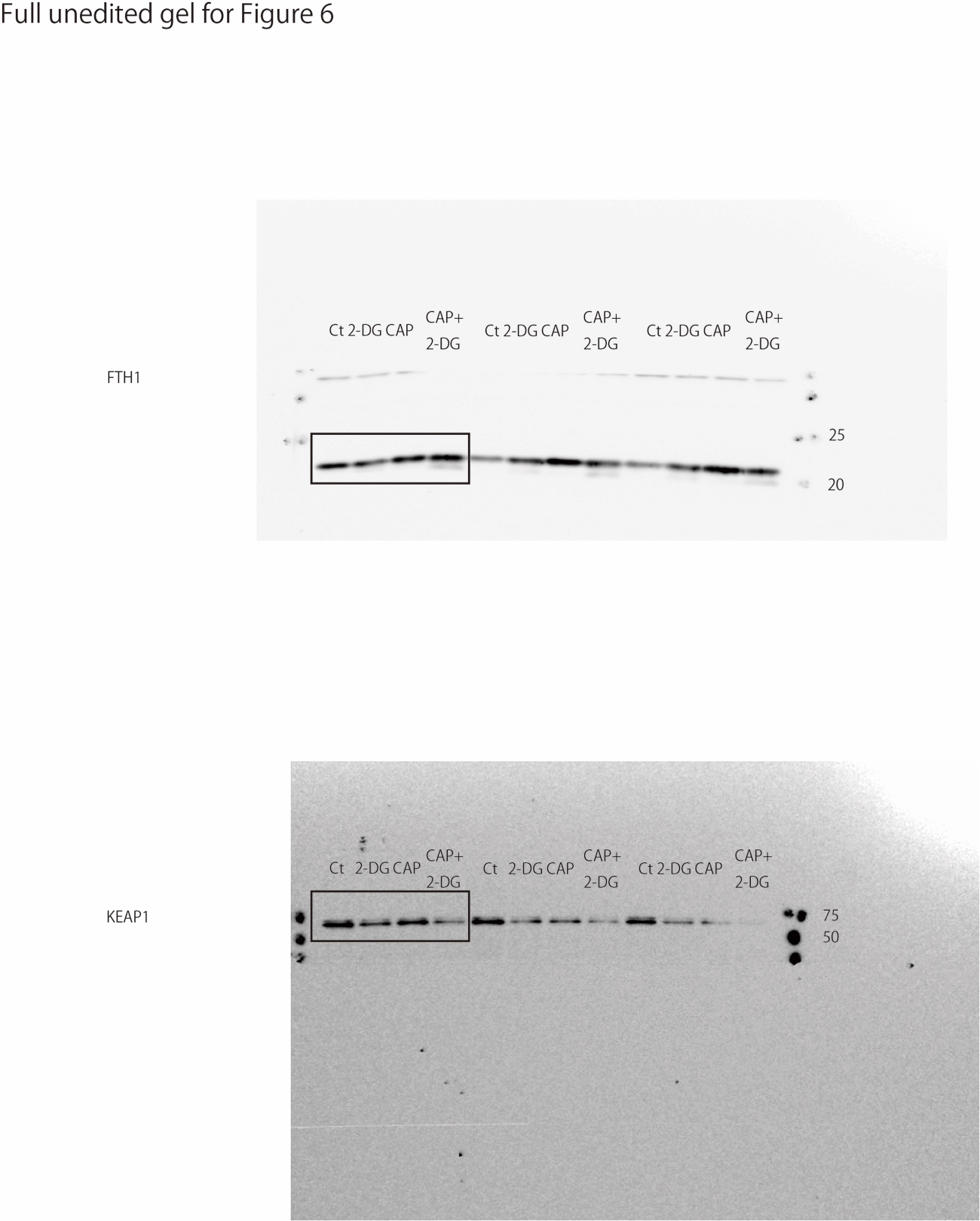


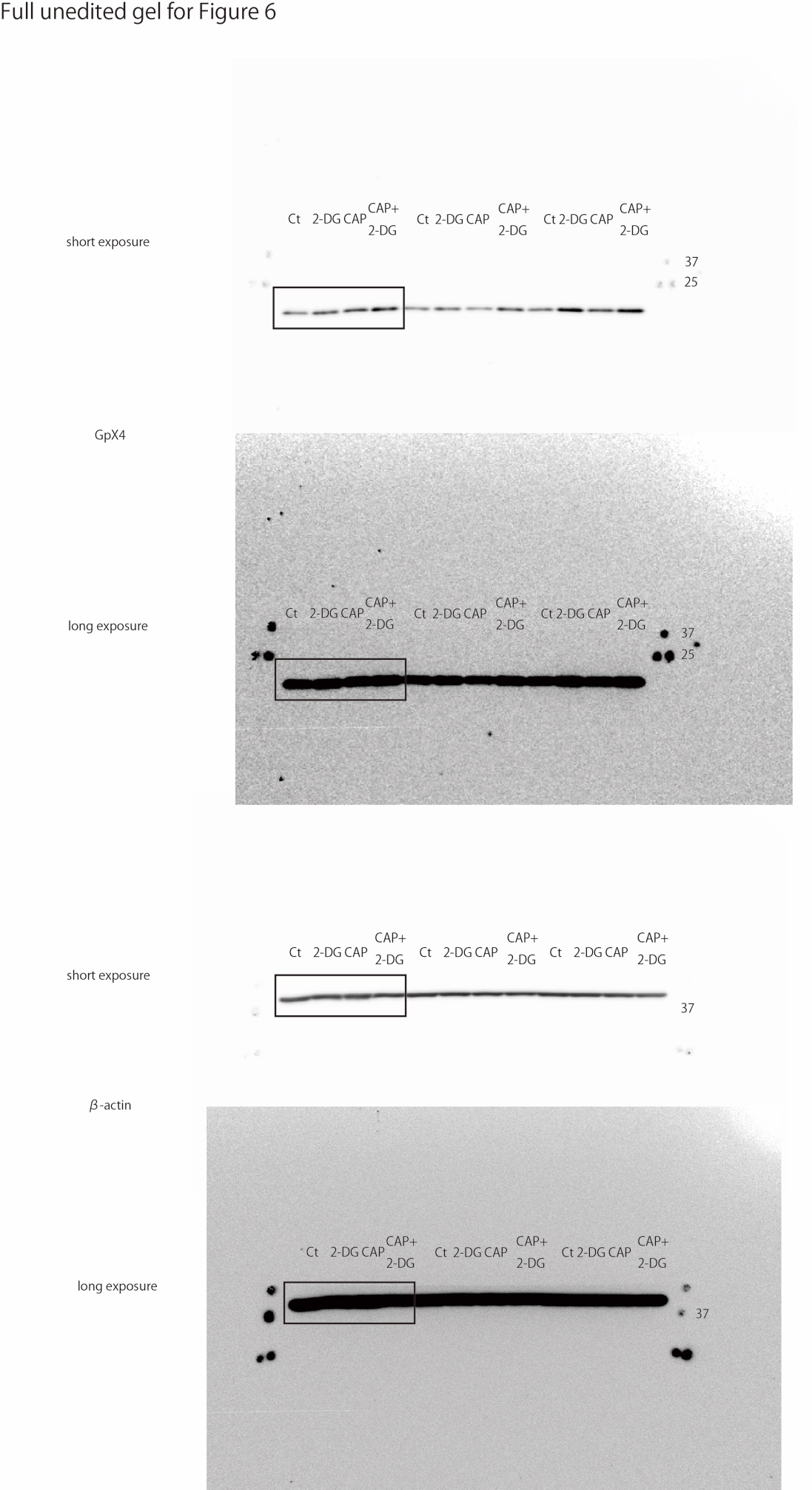


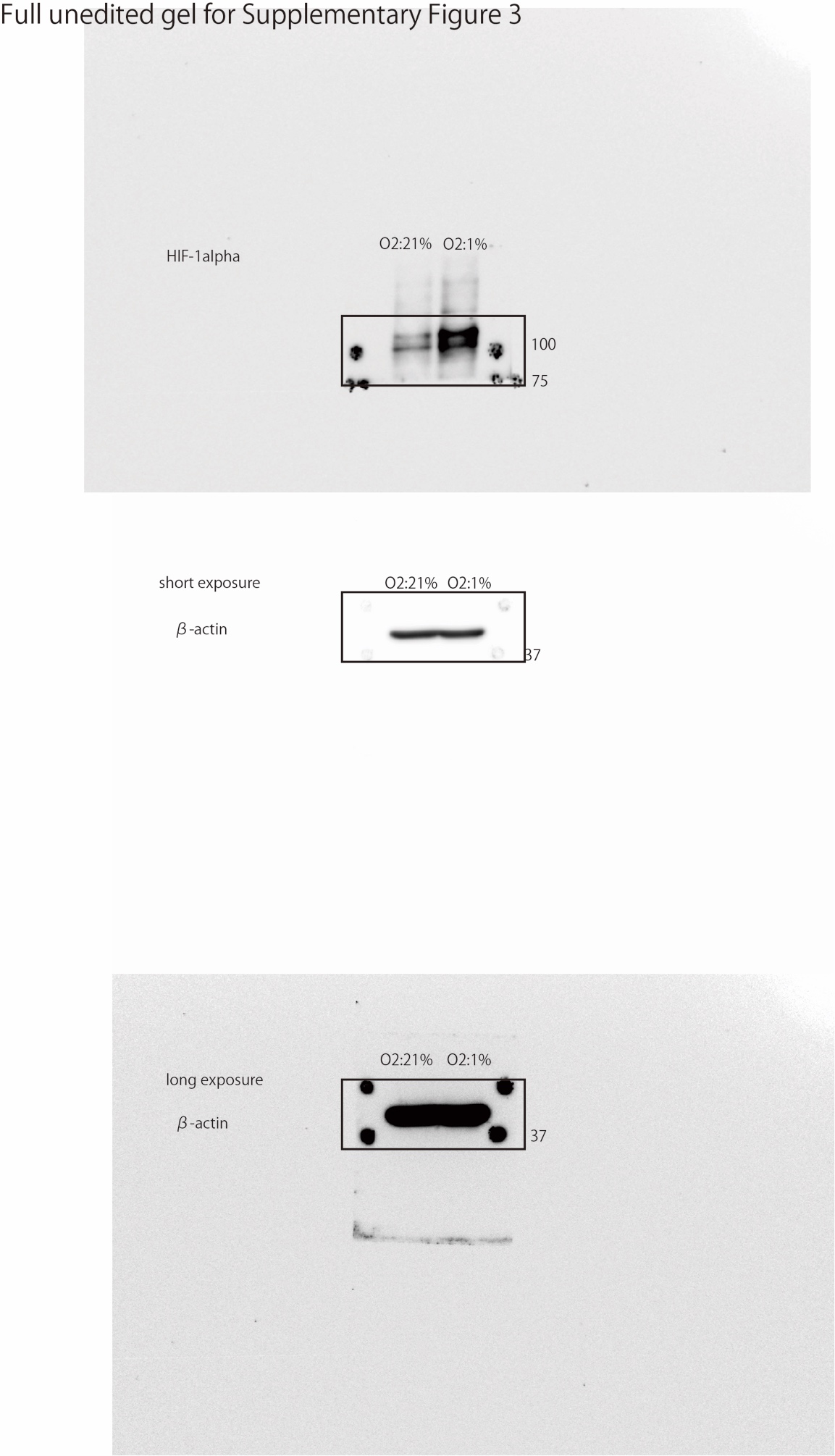


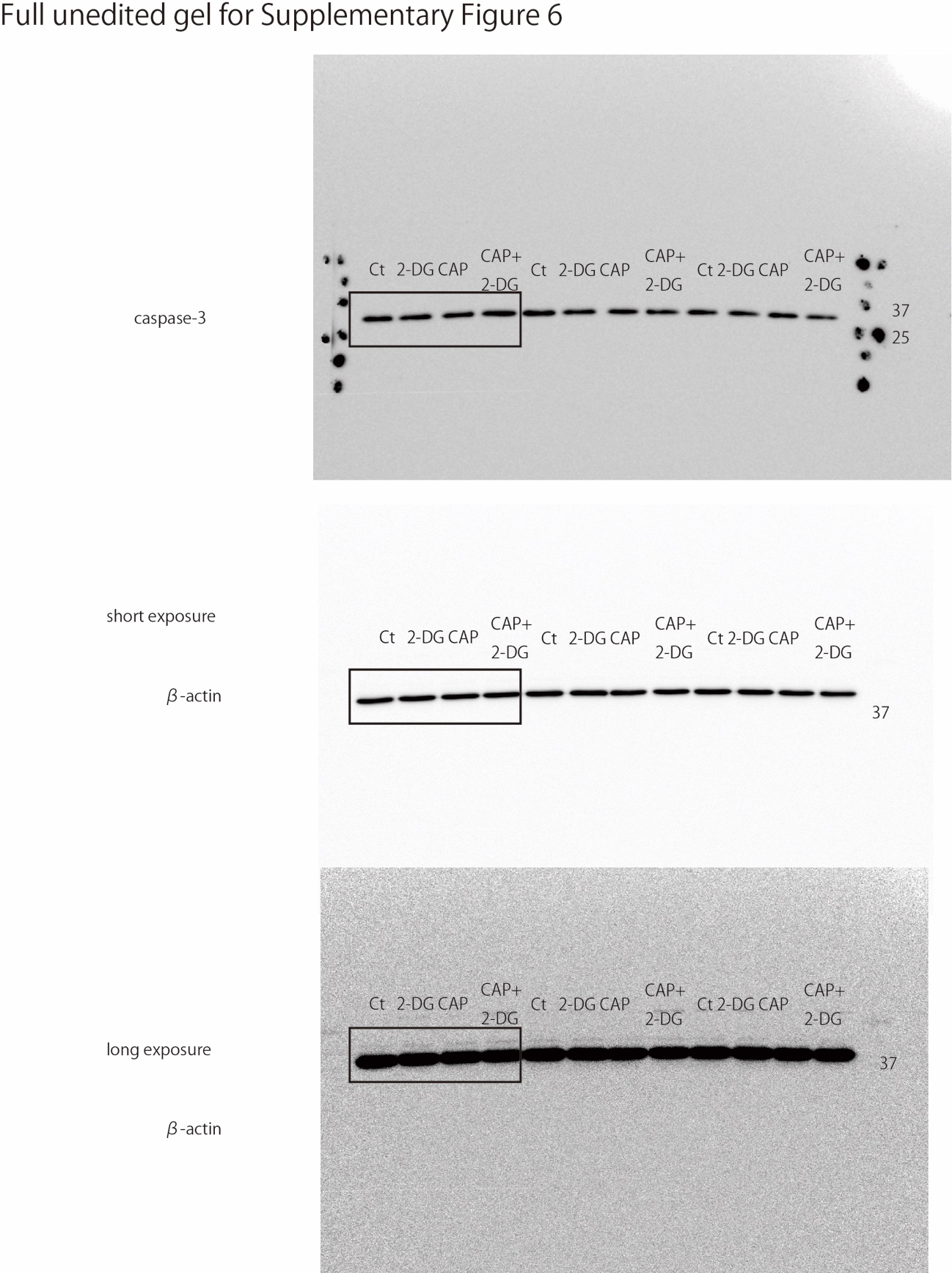

Supplement: Supplementary file 1 — Supplementary Information. [file 41598_2023_37483_MOESM1_ESM.docx]
